# The safety and efficacy of Baricitinib for systemic lupus erythematosus: a systematic review and meta-analysis of randomized controlled trials

**DOI:** 10.1097/MS9.0000000000002548

**Published:** 2024-09-05

**Authors:** Alaa Ramadan, Ibrahim Gowaily, Othman Saleh, Mohamed Abuelazm, Unaiza Ahmad, Mohammad A. Elzeftawy, Kengo Nathan Ezie, Basel Abdelazeem

**Affiliations:** a Faculty of Medicine, South Valley University, Qena; b Faculty of Medicine, Tanta University, Tanta; c Faculty of Medicine, The Hashemite University, Zarqa, Jordan; d Faculty of Medicine, Faisalabad Medical University, Faisalabad, Pakistan; e Faculty of Medicine and Biomedical Sciences of Garoua, University of Garoua, Garoua Cameroon; f West Virginia University, Morgantown, WV, USA

**Keywords:** Baricitinib, glucocorticoid, lupus, meta-analysis, SLE, systematic review

## Abstract

**Background and objective:**

Baricitinib is a JAK1 and JAK2 inhibitor approved for treating active rheumatoid arthritis and atopic dermatitis. Therefore, the authors aim to evaluate the safety and efficacy of once-daily oral Baricitinib 2 mg or 4 mg versus placebo in active SLE patients receiving standard care.

**Methods:**

The authors synthesized randomized controlled studies (RCTs) from MEDLINE, Scopus, EMBASE, PubMed, and Cochrane Library until 20 March 2023. The study protocol was registered in PROSPERO.

**Results:**

Three RCTs with 1849 participants were included. The Baricitinib group had a significant SRI-4 response [RR: 1.11 with 95% CI (1.03, 1.21), *P*=0.008] and greater than or equal to 4-point SLEDAI-2K domain improvement [RR: 1.13 with 95% CI (1.02, 1.25), *P*=0.02] compared to the placebo group; however, there was no statistically significant difference between the two groups, regarding the secondary endpoints. For safety outcomes, Baricitinib was significantly associated with a higher incidence of Any serious adverse event [RR: 1.48 with 95% CI (1.07, 2.05), *P*=0.02].

**Conclusion:**

Baricitinib is associated with significant outcomes of SRI-4 response, greater than or equal to 4-point improvement SLEDAI-2K score, and Joint Indices. Regarding safety, there was no difference in the outcomes other than the serious adverse events.

## Introduction

Highlights

- Baricitinib is associated with a significant outcome of SRI-4 response.
- Regarding safety, no difference in the outcomes other than the serious adverse events.
- Further studies are required before making definitive claims.

Systemic lupus erythematosus (SLE) is an autoimmune disorder that impacts 3.17 million people worldwide^1^. Middle-aged females are the major population, with a staggering 10:1 female-to-male ratio. SLE is a relapsing-remitting disease characterized by extensive immunological dysregulation, multiple organ inflammation, and excessive production of autoantibodies against self-antigens^2^. Multiple factors, including the environment, incidental, and genetic predisposition, contribute to SLE development^3^.

Current treatment modalities, including immunosuppressive medications, glucocorticoids, and antimalarials, though guided by EULAR recommendations, often fall short in providing sustained disease control, preventing flare-ups, and improving patients’ quality of life^4^, The development of new therapies has been slow, with few drugs receiving FDA approval in the past six decades. However, recent advancements in understanding the molecular mechanisms underlying SLE have shifted the therapeutic focus toward biologically targeted treatments. Key among these are human monoclonal antibodies like Belimumab and Anifrolumab, which have shown promise in modulating the immune response^5^.

The pathophysiology of SLE includes a wide spectrum of abnormalities induced by the dysregulation of innate and acquired immune reactions. The complement system, polymorphonuclear leukocytes monocytes, and auto-reactive T and B lymphocytes are all involved^3,6,7^. Multiple cytokines are involved in SLE pathogenesis^8,9^. The cytokines type I interferon (IFN), type II IFN, IL-6, IL-12/23, IL-17, and B lymphocyte stimulator (BAFF/BlyS) activate the Janus kinases (JAKs), a class of intracellular non-receptor tyrosine kinases^8–11^. The binding of cytokines induces a structural shift in the receptor allowing the autophosphorylation of JAKs. This initiates a signaling cascade resulting in the phosphorylation of downstream elements known as STATs (signal transducer and activator of transcription). Activated STATs disassociate from cytokine receptors and dimerize, eventually migrating to the nucleus where they regulate gene expression^12,13^.

Baricitinib is an oral, specific, and reversible JAK1 and JAK2 inhibitor authorized for the management of rheumatoid arthritis, atopic dermatitis, and severe alopecia areata in many countries^14,15^. Baricitinib has been found to considerably lower the amount of anti-dsDNA antibodies^16^, cytokines associated with the JAK-STAT pathway, IFNs, and many interleukins, such as IL-6^17,18^. In phase II randomized controlled trial (RCT), oral Baricitinib (4 mg) combined with standard of care (SOC) was more efficacious than the control arm at ameliorating SLE disease severity at week 24^19^. Moreover, recent phase III RCTs: “SLE-BRAVE-I and II,” have assessed the efficacy and safety profile of Baricitinib 2 mg and 4 mg in active lupus patients^20,21^. Only SLE-BRAVE-I was successful in achieving SLE Responder Index-4 (SRI-4) at 52 weeks^20^. Hence, the evidence of Baricitinib efficacy in SLE is still ambiguous. This paper attempts to further explore the controversy by aggregating the available data and analyzing the safety and efficacy of once-daily oral baricitinib 2 mg and 4 mg at 24 weeks and 52 weeks as opposed to placebo in SLE patients receiving SOC.

## Methodology

### Protocol registration

The methodological plan of this systematic review and meta-analysis was registered and published on PROSPERO with ID number: CRD42023413579. We followed the guidelines of Preferred Reporting Items for Systematic Reviews and Meta-Analyses (PRISMA) statement^21^ and the Cochrane Handbook of Systematic Reviews and meta-analysis^22^. (Table S1, Supplemental Digital Content 1, http://links.lww.com/MS9/A599).

The work has been reported in line with AMSTAR, Supplemental Digital Content 2, http://links.lww.com/MS9/A600 (Assessing the methodological quality of systematic reviews) Guidelines^23^. Our review achieved a high level of compliance with AMSTAR 2 criteria.

### Data sources and search strategy

Two reviewers (M.A. and B.A.) independently conducted an electronic systematic search across multiple databases, including PubMed (MEDLINE), EMBASE, Web of Science, SCOPUS, and the Cochrane Central Register of Controlled Trials (CENTRAL). The search spanned from inception until 21 March 2023, without applying any search limits. The comprehensive search strategy aimed to capture all relevant studies by including a combination of keywords and MeSH terms related to “Systemic Lupus Erythematosus,” “SLE,” “Baricitinib,” “randomized controlled trials,” “clinical trials,” and relevant synonyms. For instance, the search terms used on PubMed included combinations like (“Systemic Lupus Erythematosus” OR “SLE”) AND (“Baricitinib” OR “JAK inhibitor”) AND (“randomized controlled trial” OR “RCT” OR “clinical trial”). The detailed search strings for each database are provided in (Table S2, Supplemental Digital Content 1, http://links.lww.com/MS9/A599). Additionally, reference lists of included studies and relevant reviews were hand-searched to ensure comprehensive coverage of the literature.

### Eligibility criteria

The inclusion criteria for the review were defined using the PICO framework:

Population (P): Patients diagnosed with SLE, without restriction on age, sex, or disease duration.

Intervention (I): Treatment with Baricitinib at doses of 2 mg or 4 mg.

Control (C): Placebo.

Outcomes (O):

Primary outcome: Achievement of SLE Responder Index-4 (SRI-4) at week 24.

Secondary outcomes: Including, but not limited to, SELENA-SLEDAI Flare Index, SLEDAI-2K remission of arthritis or rash, Lupus Low Disease Activity State (LLDAS), 28-tender joint count, 28-swollen joint count, and various lupus activity and treatment response scoring criteria such as the SLEDAI-2K, Physician’s Global Assessment (PGA), and Cutaneous Lupus Erythematosus Disease Area and Severity Index (CLASI)-Activity Score.

The review also considered safety outcomes, including the incidence of any adverse event, serious adverse events, infections, serious infections, herpes zoster virus, and deep-vein thrombosis.

Exclusion criteria were:

Non-randomized studies, observational studies, case reports, and reviews.

Studies not involving Baricitinib or not using the specified doses (2 mg or 4 mg).

Trials involving populations other than SLE patients or not reporting the specified primary and secondary outcomes.

### Selection process

The selection process involved two phases: initial screening of titles and abstracts followed by full-text review. M.A.E. and I.G. independently screened titles and abstracts using Covidence online software, excluding duplicates and irrelevant studies. The full-text screening was conducted by the same reviewers, adhering to the eligibility criteria outlined above. Discrepancies between reviewers were resolved through discussion. In cases where consensus could not be reached, a third reviewer (B.A.) was consulted to provide a final decision. This rigorous selection process aimed to minimize bias and ensure the inclusion of high-quality studies.

During data extraction and risk of bias assessment, any disagreements were similarly addressed through consensus or by consulting the third reviewer. This method ensured consistency and accuracy in data collection and evaluation, contributing to the reliability and validity of the systematic review findings.

### Data extraction

A previously pilot-tested extraction Excel sheet was used by (M.A.E. and I.G.) to extract the following data from the included studies: summary characteristics (first author name, year of publication, country, study design, total participants, Baricitinib dose, duration of treatment, follow-up duration, inclusion criteria and, primary outcome); baseline information (number of patients in each group, age, sex, SLEDAI-2K score, SLEDAI-2K score greater than or equal to 10, physician’s global assessment score, CLASI-activity score, tender joint count, swollen joint count, SLICC/ACR damage index score, and concomitant medication); study outcomes (SRI-4 at week 24, adverse events, SELENA-SLEDAI flare index, SLEDAI-2K remission of arthritis or rash, LLDAS, 28-tender joint count, and 28-swollen joint count). Conflicts were discussed and resolved by consensus.

### Quality assessment

Two independent investigators (M.A.E. and I.G.) implemented the revised Cochrane Collaboration’s tool for assessing the risk of bias in RCTs (ROB 2)^24^, considering selection, performance, detection bias, attrition, reporting, and other potential sources of biases. Any disagreement was resolved through discussion or by a third reviewer (B.A.). Furthermore, two independent investigators (M.A. and B.A.) used the GRADE (Grading of Recommendations, Assessment, Development, and Evaluation) approach to evaluate the quality of the evidence^25^.

### Statistical analysis

This meta-analysis was conducted using Revman software version 5.4^26^ to pool dichotomous outcomes using risk ratio (RR) and continuous outcomes using standardized mean difference (SMD), along with the corresponding 95% CI. Pooled analysis was conducted using the fixed-effects model; however, the random-effects model was implemented in case of significant heterogeneity. Heterogeneity was evaluated using the χ^2^ test and measured using the I^2^ test. The χ^2^ test was considered significant on an alpha level below 0.1, and heterogeneity was considered significant if the I^2^ was greater than 50%. On significant heterogeneity, sensitivity analysis by excluding one study at a time and rerunning the analysis was conducted to investigate the source of heterogeneity.

## Results

### Search results and study selection

**Figure 1:**
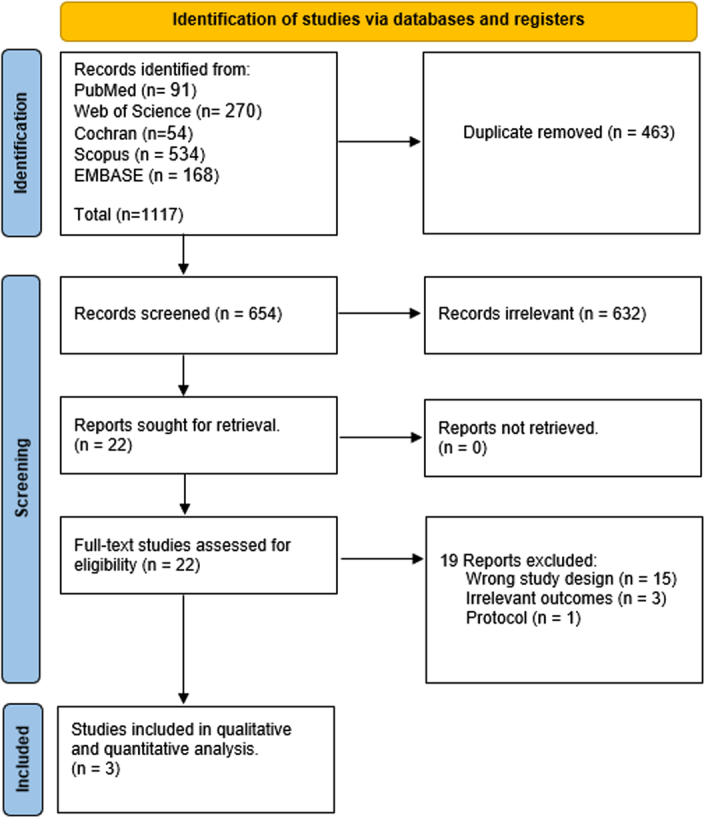
Preferred Reporting Items for Systematic Reviews and Meta-Analyses (PRISMA) flow chart of the screening process.

The search process identified 1117 studies, which were evaluated based on their titles and abstracts. Following the exclusion of duplicates (463) and irrelevant studies (632), twenty-two full-text articles were reviewed. Finally, three studies were included in qualitative and quantitative synthesis (Fig. 1).

### Characteristics of included studies

We included three RCTs with a total of 1849 patients^19–21^. The majority of participants were female. The mean age of the intervention group across the three studies ranged from 41.5 to 45 years old. The treatment duration was extended to 52 weeks in two trials^20,21^, while in the third study, it lasted for 24 weeks^19^. A detailed summary and baseline characteristics of the included studies are illustrated in (Tables 1 and 2).

**Table 1:** Summary characteristics of the included studies

References	Study design	Total participants	No. centers	Duration therapy	Main inclusion criteria	Primary outcome
Petri *et al.* (BRAVE-1)^21^	Double-blinded, multicenter phase III RCT	775	162	52 weeks	≥18 years or older, diagnosed clinically and with laboratory tests with SLE (active disease)	(SRI)-4 response at week 52
Morand *et al.* (BRAVE-2)^20^	Double-blinded, multicenter phase III RCT	760	182	52 weeks	≥18 years or older, diagnosed clinically and with laboratory tests with SLE (active disease)	(SRI)-4 response at week 52
Wallace *et al.* (RAPID)^19^	Double-blinded, multicenter phase II RCT	314	78	24 weeks	≥18 years or older, diagnosed clinically and with laboratory tests with SLE (active disease)	SLEDAI-2K, at week 24.

RCT, randomized controlled trial; SLE, systemic lupus erythematosus; SLEDAI-2K, Systemic Lupus Erythematosus Disease Activity Index-2000; SRI-4, SLE Response Index-4.

**Table 2:** Baseline characteristics of the included studies

References	No. patients in each group		Sex (male), *N* (%)		Age (year), mean (SD)		SLEDAI-2K score, mean (SD)		Physician’s global assessment score, mean (SD)		CLASI-activity score, mean (SD)		Tender joint count, mean (SD)		Swollen Joint Count, mean (SD)		SLICC/ACR damage index score, mean (SD)	
	Baricitinib	Placebo	Barcitinib (4/2 mg)	Placebo	Barcitinib (4/2 mg)	Placebo	Barcitinib (4/2 mg)	Placebo	Barcitinib (4/2 mg)	Placebo	Barcitinib (4/2 mg)	Placebo	Barcitinib (4/2 mg)	Placebo	Barcitinib (4/2 mg)	Placebo	Barcitinib (4/2 mg)	Placebo
Petri *et al.* (BRAVE-1)^21^	4 mg=258, 2 mg =261	256	13 (5)/15 (6)	15 (6)	42.2 (12.1)/ 42.8 (13.0)	43.5 (13.5)	10.1 (3.0)/ 10.1 (3.4)	10.1 (3.2)	58.8 (14.6)/ 61.0 (12.5)	60.0 (14.6)	6.7 (5.8)/6.6 (6.6)	6.8 (6.1)	10.6 (7.1)/ 10.3 (6.8)	10.6 (7.1)	6.68 (5.0)/ 6.64 (5.1)	6.41 (5.4)	0.60 (1.0)/ 0.68 (1.1)	0.66 (1.1)
Morand *et al.* (BRAVE-2)^20^	4 mg=252, 2 mg=255	253	15 (6)/17 (7)	16 (6)	41.5 (12.9)/ 42.9 (12.4)	42.0 (12.0)	10.0 (3)/ 10.3 (3)	10.1 (3)	1.8 (0.5)/1.8 (0.4)	1.8 (0.5)	6.1 (5.6)/6.5 (7.0)	6.3 (6.2)	10.9 (7.0)/ 10.3 (6.5)	10.0 (7.0)	6.9 (5.4)/ 6.9 (5.1)	7.1 (5.6)	0.6 (1.0)/ 0.6 (1.0)	0.6 (1.0)
Wallace *et al.* (RAPID)^19^	4 mg=104, 2 mg=105	105	5 (4.8)/9 (8.6)	6 (5.7)	45.0 (12.4)/ 43.2 (11.0)	44.9 (12.8)	9.0 (3.3)/ 8.8 (3.4)	8.9 (2.9)	51.7 (16.0)/ 48.8 (15.8)	49.5 (16.9)	4.0 (3.4)/3.8 (5.4)	4.9 (5.7)	8.5 (6.2)/ 8.7 (6.6)	7.7 (5.8)	5.5 (4.2)/ 5.2 (4.7)	5.3 (4.7)	0.40 (0.88)/ 0.44 (0.68)	0.59 (0.97)

CLASI, Cutaneous Lupus Erythematosus Disease Area and Severity Index; SLEDAI-2K, Systemic Lupus Erythematosus Disease Activity Index-2000.

### Risk of bias and quality of evidence

**Figure 2:**
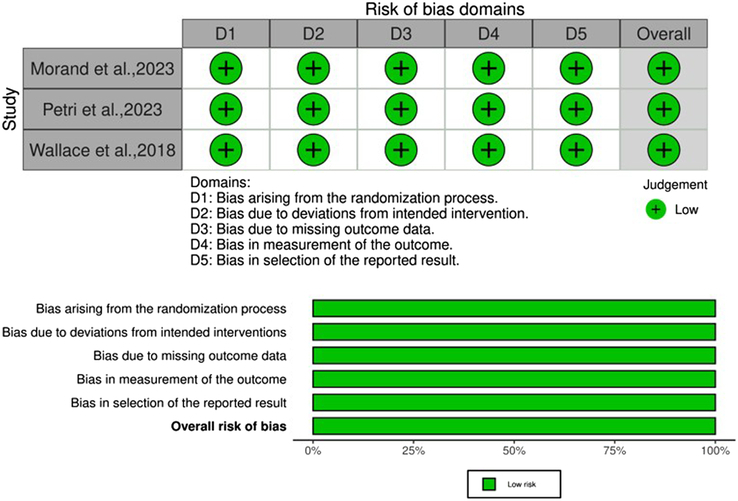
Quality assessment of the risk of bias in the included trials.

The findings of the quality assessment of the studies are presented in (Fig. 2).

### Efficacy outcomes

#### Lupus erythematosus responder index-4 (SRI-4)

**Figure 3:**
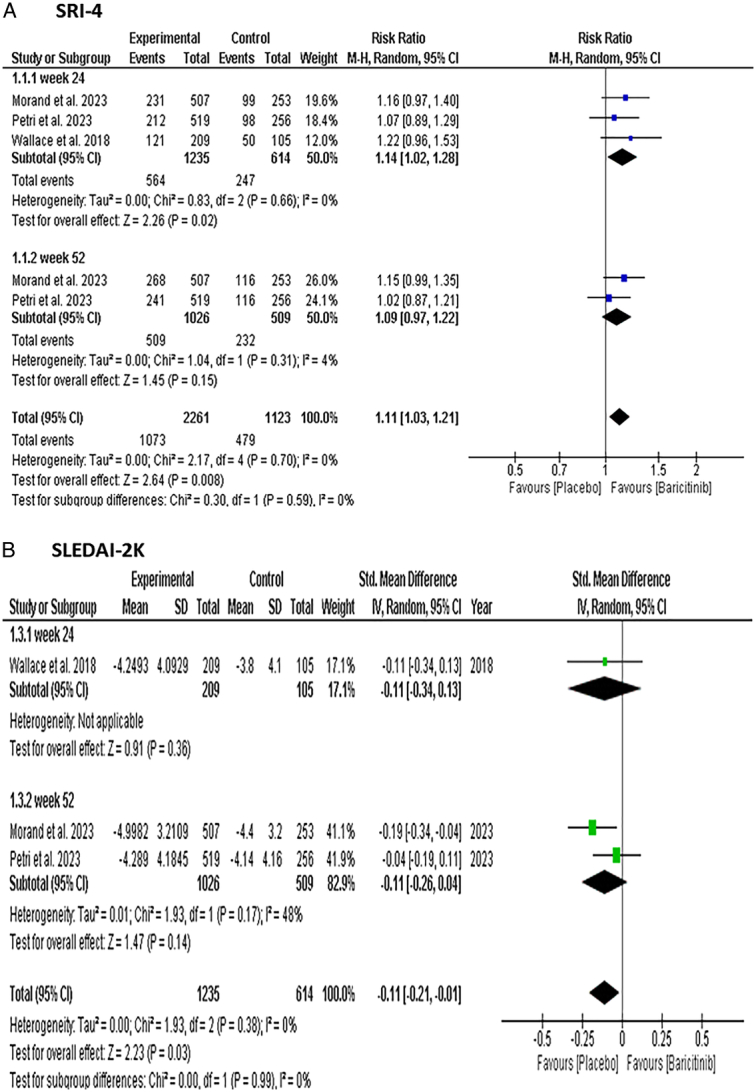
Forest plot of the efficacy outcomes [(A) SRI-4 response], [(B) SLEDAI-2K score]. SLEDAI-2K, Systemic Lupus Erythematosus Disease Activity Index-2000; SRI-4, SLE Responder Index-4.

Our analysis showed that the Baricitinib group had a statistically significant SRI-4 response than the placebo group [RR: 1.11 with 95% CI (1.03, 1.21), *P*=0.008] (Fig. 3-A). A subgroup analysis revealed that the proportion of patients in the Baricitinib group who achieved an SRI-4 response at week 24 was higher than that in the placebo group [RR: 1.14 with 95% CI (1.02, 1.28), *P*=0.02], while there was no significant difference between the two groups at week 52 [RR: 1.09 with 95% CI (0.97, 1.22), *P*=0.15] (Fig. 3-A). Moreover, a network meta-analysis was used to provide evidence of comparative effectiveness between the Baricitinib doses. After 24 weeks, the analysis found that Baricitinib in a 4 mg dose was significantly associated with a larger (SRI)-4 response than those with a 2 mg dose [RR: 1.20 with 95% CI (1.05, 1.36)] (Figure S1, Supplemental Digital Content 1, http://links.lww.com/MS9/A599).

#### Systemic lupus erythematosus disease activity index-2000 (SLEDAI-2 K) score

The analysis showed that the placebo group was associated with a significant SLEDAI-2K score reduction compared to the Baricitinib group [MD: −0.11 with 95% CI (−0.21, −0.01), *P*=0.03] (Fig. 3-B). The subgroup analysis based on different time frames; 24 weeks [MD: −0.11 with 95% CI (−0.34, −0.13), *P*=0.36] or 52 weeks [MD: −0.11 with 95% CI (−0.26, −0.04), *P*=0.14], did not show a significant improvement in the score (Fig. 3-B).

#### (SLEDAI-2 K) remission of arthritis or rash

**Figure 4:**
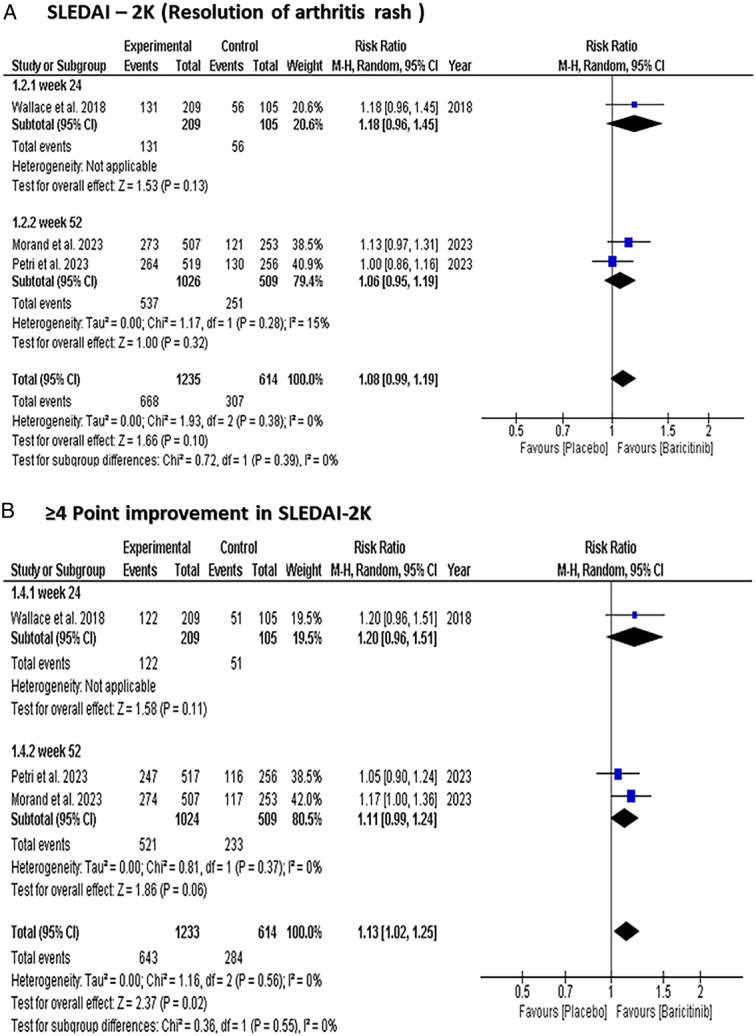
Forest plot of the efficacy outcomes [(A) SLEDAI-2K (Resolution of arthritis/rash)], [(B) ≥4-point improvement in SLEDAI-2K domain]. SLEDAI-2K, Systemic Lupus Erythematosus Disease Activity Index-2000.

There was no significant difference in SLEDAI-2K remission of arthritis or rash between both groups [RR: 1.08 with 95% CI (0.99, 1.19), *P*=0.10] (Fig. 4-A). The subgroup analysis did not show a significant difference in the score either at 24 weeks [RR: 1.18 with 95% CI (0.96, 1.45), *P*=0.13] or 52 weeks [RR: 1.06 with 95% CI (0.95, 1.19), *P*=0.32].

#### Reduction of ≥4 points from baseline in (SLEDAI-2 K) score

For participants who received Baricitinib, a significant reduction of ≥4 points from baseline in SLEDAI-2 K score over placebo was observed [RR: 1.13 with 95% CI (1.02, 1.25), *P*=0.02] (Fig. 4-B). However, the subgroup analysis based on time did not show a significant reduction between both groups either at 24 weeks [RR: 1.20 with 95% CI (0.96, 1.51), *P*=0.11] or 52 weeks [RR: 1.11 with 95% CI (0.99, 1.24), *P*=0.06].

#### No worsening by PGA

**Figure 5:**
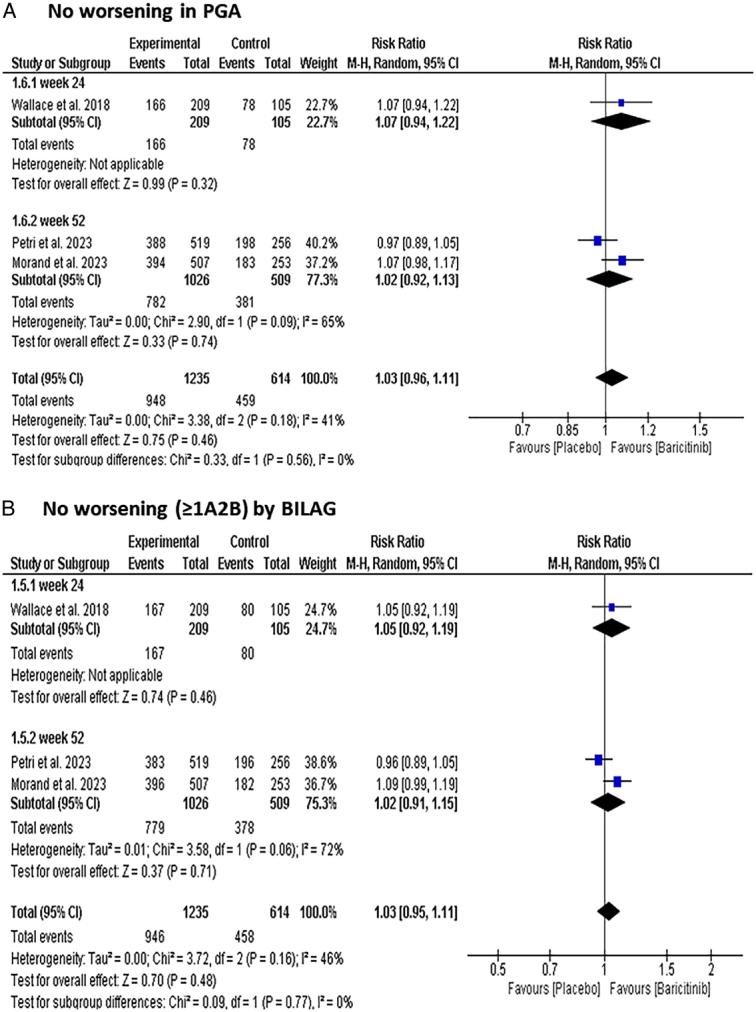
Forest plot of the efficacy outcomes [(A) No worsening in PGA, (B) no worsening (≥1A/2B) by BILAG]. BILAG, British Isles Lupus Assessment Group; PGA, Physician’s Global Assessment.

There was no significant change regarding no worsening in PGA [RR: 1.03 with 95% CI (0.96, 1.11), *P*=0.48] (Fig. 5-A). Also, the subgroup analysis did not show a significant difference between both groups at 24 weeks [RR: 1.07 with 95% CI (0.94, 1.22), *P*=0.32] and at 52 weeks [RR: 1.02 with 95% CI (0.92, 1.13), *P*=0.74].

#### No new British Isles Lupus Assessment Group (BILAG) A and BILAG B score

Regarding BILAG, there was no significant difference between the Baricitinib group and the placebo group [RR: 1.03 with 95% CI (0.95, 1.11), *P*=0.48] (Fig. 5-B). The subgroup analysis did not show a significant difference between both groups at 24 weeks [RR: 1.05 with 95% CI (0.92, 1.19), *P*=0.46] and at 52 weeks [RR: 1.02 with 95% CI (0.91, 1.15), *P*=0.71].

#### Joint pain

There was no significant difference between the Baricitinib group and the placebo group regarding worst pain NRS [MD: −0.07 with 95% CI (−0.16, 0.03), *P*=0.17] (Figure S2, Supplemental Digital Content 1, http://links.lww.com/MS9/A599), worst joint pain NRS [MD: −0.08 with 95% CI (−0.20, 0.04), *P*=0.21] (Figure S3, Supplemental Digital Content 1, http://links.lww.com/MS9/A599), 28-tender joint count [MD: −0.12 with 95% CI (−0.25, 0.00), *P*=0.06] (Figure S4, Supplemental Digital Content 1, http://links.lww.com/MS9/A599), and 28-swollen joint count [MD: −0.08 with 95% CI (−0.18, 0.02), *P*=0.10] (Figure S5, Supplemental Digital Content 1, http://links.lww.com/MS9/A599).

#### Heterogeneity

The pooled studies were homogenous in SRI-4 response (I^2^=0%, *P*=0.70), SLEDAI-2K (*P*=0.38, I^2^=0%), no worsening in PGA (*P*=0.18, I^2^=41%), no worsening (≥1A/2B) by BILAG (*P*=0.16, I^2^=46%), greater than or equal to 4-point improvement in SLEDAI-2K (*P*=0.56, I^2^=0%), Worst Pain NRS (*P*=0.42, I^2^=0%), worst joint pain NRS (*P*=0.22, I^2^=35%), SLEDAI-2K (resolution of arthritis/rash) (*P*=0.38, I^2^=0%), 28-tender joint count (*P*=0.19, I^2^=39%), and 28-swollen joint count (*P*=0.34, I^2^=7%).

#### Other subgroup analyses

Considering the subgroup analysis for worst joint pain NRS, we found a significant early improvement (after 24 weeks) in the Baricitinib group [MD: −0.26 with 95% CI (−0.50, −0.03), *P*=0.03]. Regarding 28-tender joint count, the Subgroup analysis showed that the Baricitinib group at week 24 had a significant outcome compared to the placebo group [MD: −0.27 with 95% CI (−0.50, −0.03), *P*=0.03], while there was no difference between the two groups after 52 weeks [MD: −0.08 with 95% CI (−0.20, 0.04), *P*=0.18] (Figure S4, Supplemental Digital Content 1, http://links.lww.com/MS9/A599). Moreover, the subgroup analysis of 28-swollen joint count showed a significant improvement in the Baricitinib group compared to the placebo group at week 52 [MD: −0.12 with 95% CI (−0.22, −0.01), *P*=0.03] (Figure S5, Supplemental Digital Content 1, http://links.lww.com/MS9/A599).

### Safety outcomes

#### Adverse events

**Figure 6:**
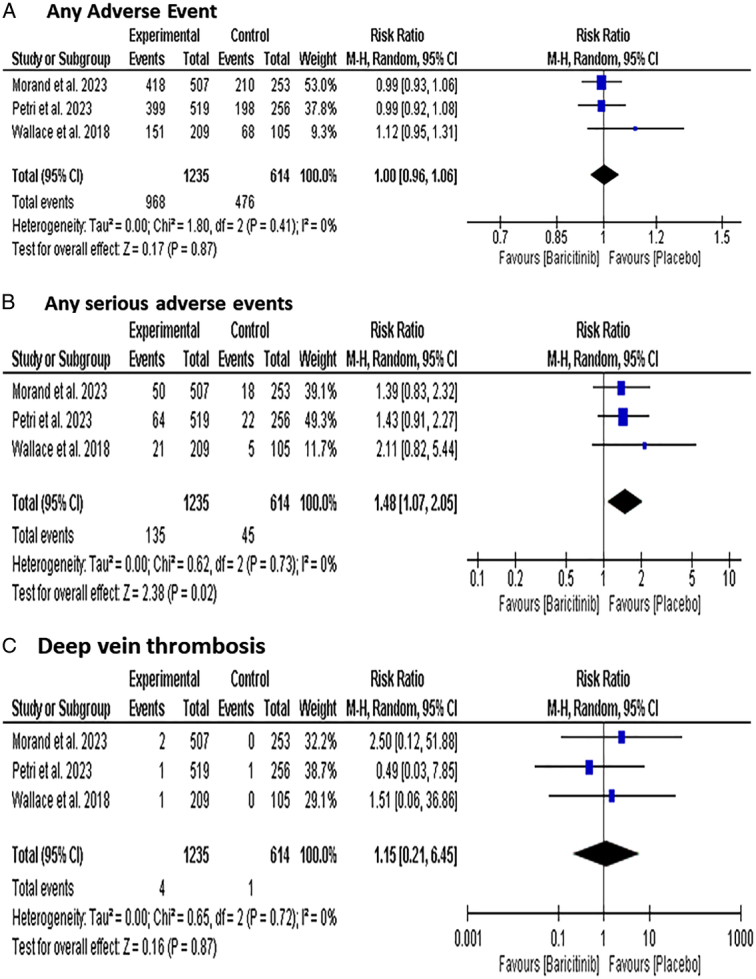
Forest plot of the safety outcomes [(A) Any adverse events, (B) any serious adverse event, (C) deep-vein thrombosis].

Baricitinib group was significantly associated with a higher incidence of Any serious adverse event [RR: 1.48 with 95% CI (1.07, 2.05), *P*=0.02] (Fig. 6-B). Moreover, the subgroup analysis showed that Baricitinib 2 mg and 4 mg significantly outperformed placebo [RR=1.49, 95% CI (1.16, 1.92), *P*=0.002] (Figure S6, Supplemental Digital Content 1, http://links.lww.com/MS9/A599).

There was no difference between the two groups regarding any adverse events [RR: 1.00 with 95% CI (0.96, 1.06), *P*=0.87] (Fig. 6-A). The subgroup analysis of any adverse events did not find a significant difference between Baricitinib in the 2 mg [RR: 1.01 with 95% CI (0.95, 1.07), *P*=0.86] dose and 4 mg dose [RR: 1.02 with 95% CI (0.96, 1.08), *P*=0.58] (Figure S7, Supplemental Digital Content 1, http://links.lww.com/MS9/A599).

#### Deep-vein thrombosis

There was no difference between the two groups regarding deep-vein thrombosis [RR: 1.15 with 95% CI (0.21, 6.45), *P*=0.87] (Fig. 6-C). The subgroup analysis did not show a significant difference between the 2 mg dose [RR: 2.30 with 95% CI (0.34, 15.51), *P*=0.36] and 4 mg dose [RR: 1.00 with 95% CI (0.14, 7.04) *P*>0.99] groups (Figure S8, Supplemental Digital Content 1, http://links.lww.com/MS9/A599).

#### Infections

**Figure 7:**
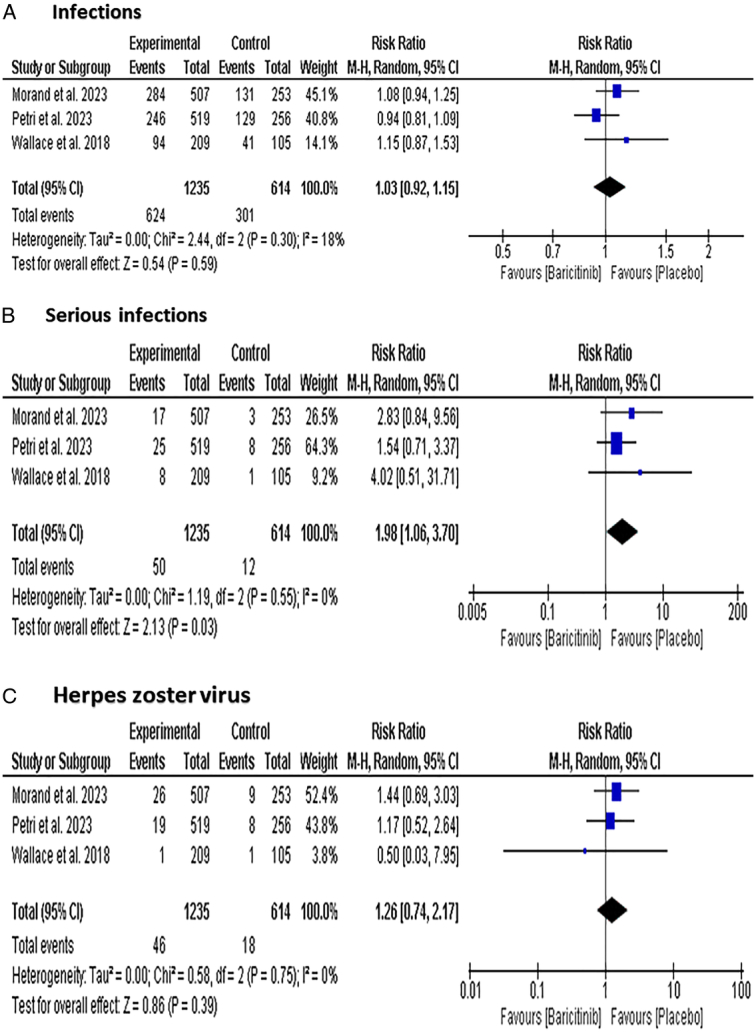
Forest plot of the safety outcomes [(A) Infections, (B) serious infections, (C) Herpes zoster virus].

In terms of Infections, there was no significant difference between the Baricitinib group and the placebo group [RR: 1.03 with 95% CI (0.92, 1.15), *P*=0.59] (Fig. 7-A). The subgroup analysis did not show a significant difference between the 2 mg dose [RR: 0.01 with 95% CI (−0.04, 0.07), *P*=0.62] and 4 mg dose [RR: 0.02 with 95% CI (−0.04, 0.08), *P*=0.57] groups (Figure S9, Supplemental Digital Content 1, http://links.lww.com/MS9/A599)

Regarding serious infections, there was no significant difference between the Baricitinib group and the placebo group [RR: 1.98 with 95% CI (1.06–3.70), *P*=0.03] (Fig. 7-B). The subgroup analysis showed the Baricitinib 4 mg dose participants had significantly more serious infections than the placebo group [RR: 2.33 with 95% CI (1.20, 4.53), *P*=0.01] (Figure S10, Supplemental Digital Content 1, http://links.lww.com/MS9/A599).

The Baricitinib group and the placebo group did not differ significantly in terms of obtaining herpes zoster virus [RR: 1.26 with 95% CI (0.74, 2.17), *P*=0.39] (Fig. 7-C). The subgroup analysis did not show a significant difference between the 2 mg dose [RR: 0.93 with 95% CI (0.49, 1.78), *P*=0.84] and 4 mg dose [RR: 1.61 with 95% CI (0.91, 2.86), *P*=0.82] groups (Figure S11, Supplemental Digital Content 1, http://links.lww.com/MS9/A599).

#### Heterogeneity

Studies were homogenous in serious infections (*P*=0.55, I^2^=0%), infections (*P*=0.30, I^2^=18%), Herpes zoster virus (*P*=0.75, I^2^=0%), and deep-vein thrombosis (*P*=0.72, I^2^=0%), any serious adverse event (*P*=0.73, I^2^=0%), and any adverse event (*P*=0.41, I^2^=0%).

## Discussion

The findings suggest that Baricitinib, particularly at the 4 mg dosage, demonstrates promising efficacy in improving disease activity and clinical outcomes in SLE patients compared to placebo. The achievement of SRI-4 at 24 weeks and favorable secondary outcomes, such as reductions in SLEDAI-2K, indicate Baricitinib’s potential as an effective therapeutic option. Additionally, the occurrence of adverse events, infections, serious infections, herpes zoster virus, and deep-vein thrombosis (DVT) did not vary statistically between the two groups. Nonetheless, the Baricitinib group had a higher likelihood of ‘serious adverse events’. Specifically, the subgroup analysis revealed that participants receiving the 4 mg dose experienced a significantly higher incidence of serious infections compared to those in the placebo group. we found a significant early improvement (after 24 weeks) in “Worst Joint Pain NRS” and “28-tender joint count” and “late improvement (after 52 weeks) for the 28-swollen joint count”. Further, our network meta-analysis revealed that 4 mg of Baricitinib was linked with a stronger (SRI)-4 response compared to placebo at week 24. Baricitinib’s mechanism of action as a JAK1 and JAK2 inhibitor differentiates it from other current treatments, such as corticosteroids, antimalarials, and biologics like Belimumab and Anifrolumab. Unlike broad-spectrum immunosuppressants, Baricitinib specifically targets the JAK-STAT pathway, which plays a crucial role in the pathogenesis of SLE by modulating cytokine signaling^27,28^.

A recent published systematic review and meta-analysis compare the safety and efficacy of Baricitinib versus placebo in SLE participants^29^. The Baricitinib group was divided into Baricitinib 2 mg and Baricitinib 4 mg. However, they did not demonstrate the outcomes based on time. Also, assessments of 28-tender joint count, 28-swollen joint count, and worst pain numeric rating scale NRS were not included.

SRI-4 has gained popularity as the primary endpoint in clinical trials as it is an important assessment tool. Unlike other indexes, it was designed to ensure that a boost in SLE disease activity is not coupled with a deterioration of other disease symptoms. It consists of three scores; SELENA-SLEDAI (Safety of Estrogens in Lupus Erythematosus: National Assessment– Systemic Lupus Erythematosus Disease Activity Index), BILAG (British Isles Lupus Assessment Group), and PGA (physician global assessment). Achieving an SRI-4 involves a SLEDAI increment of 4 points or more, PGA not deteriorating by 0.3 points or more (10% or more), and no new BILAG A or no more than one new BILAG B domain score^30^. Data analysis from the BLISS-SC study has illustrated the value of achieving an SRI response. It demonstrates global improvements in SRI responders not only in defined outcomes but also in other clinical and laboratory measures such as a cutback in prednisone dose to less than or equal to 7.5 mg/d, diminished flare rates, ameliorated serological markers, and Functional Assessment of Chronic Illness Therapy-Fatigue score (FACIT-F) besides definition outcomes^27^. Moreover, a meta-analysis by Zhang and colleagues, based on seven randomized controlled trials (RCTs) and 19 case series, comparing Belimumab with placebo, reported that Belimumab therapy resulted in a stronger SRI-4 response rate [52.8% vs. 41.6%] and a significant 4-point reduction in the SELENA-SLEDAI score [52.0% vs. 41.3%]^28^. Therefore, additional studies in clinical settings must be conducted before Baricitinib could possibly be included in standard clinical care for SLE in the future.

Regarding the other measures of SRI-4 response, our study, however, found no difference between Baricitinib and placebo in No worsening in PGA and No worsening (≥1A/2B) by BILAG. It is important to emphasize that PGA is an essential measure for calculating SRI response since it assures that the improvement in SLEDAI score is not achieved at the cost of deterioration of patients’ overall state. Additionally, BILAG provides an accurate assessment of flare-ups as it evaluates temporal variation in SLE disease activity in distinctive organs. It was designed with the doctrine of intention to treat, that is an emphasis on clinical meaningfulness enabling decision-making to add or subtract new therapy^31,32^. Despite being a crucial component of the SRI response, conventional BILAG is an out-of-date measure, which may explain why our results were not statistically significant. Updated versions, such as BILAG-2004, are known to distinguish between responders and non-responders more effectively^33^.

Regarding subgroup analysis, we found significant results at week 24 for Worst Joint Pain NRS and 28-tender joint count and at week 52 for the 28-swollen joint count. From the perspective of the patient, symptom alleviation of fatigue and joint pain are essential in determining the drug’s effectiveness. Joint pain in SLE patients results from tenosynovitis, and the cytokine IL-6 has been associated with “the worst joint pain”^18,34^. Baricitinib downregulates both IL-6 and TNFRSF9 (The TNF receptor superfamily member 9), also called CD137 or 4-1BB. The transcription of TNFRSF9 causes T and Natural Killer (NK) cells to become activated, release inflammatory cytokines, and support antibody-dependent cellular cytotoxicity^35^. The latter is responsible for tender and swollen joints^18^. Therefore, this establishes Baricitinib as having both anti-inflammatory and analgesic properties.

### Safety

While our study highlights the potential efficacy of Baricitinib as demonstrated by significant improvements in primary endpoints, it is essential to carefully consider the safety profile of this medication. The study demonstrated a higher risk of serious infection for the 4 mg dose participants. Specifically, the subgroup analysis revealed that those receiving the 4 mg dose experienced significantly more serious infections compared to the placebo group. The observed increase in serious adverse events underscores the need for thorough risk-benefit assessments when considering Baricitinib as a treatment option for active SLE patients. These SAEs include an increased risk of infections, such as herpes zoster virus, and thromboembolic events like deep-vein thrombosis. The heightened risk may be attributed to the immunosuppressive effects of JAK inhibition, which can impair the body’s ability to mount an effective immune response. This immunosuppressive effect, while beneficial in reducing inflammation, may predispose patients to infections and other complications. Clinicians must weigh the benefits of Baricitinib’s efficacy in controlling disease activity against the potential risks of SAEs. This balance is crucial in clinical decision-making, particularly for patients with a high baseline risk of infections or thromboembolic events. The decision to use Baricitinib may require careful patient selection, considering factors such as comorbidities, prior treatment history, and individual risk profiles^36,37^.

The safety profile of Baricitinib is consistent with previously reported literature. “Any serious adverse event” had a significant relative risk for Baricitinib compared to placebo in our study. The likelihood of serious adverse events (such as mortality, serious infections including COVID-19, major adverse cardiovascular events (MACE), and malignancies) is likely to be concerning primarily in predisposed individuals. The existence of risk factors, for instance, old age, coexisting illnesses, concurrent drugs, or other variables independent of therapy, have an association with the occurrence of significant adverse events^36^. Moreover, a decrease in immune function due to various factors, including comorbidities that are common in SLE patients and using glucocorticoids and immunosuppressive regimens, have all been linked to an elevated risk of infections, particularly serious infections and herpes zoster^37,38^. However, our study did not demonstrate any increased risk of infection, serious infection, herpes zoster virus, or deep-vein thrombosis.

It is worth discussing here that our findings indicate Baricitinib 4 mg to be numerically superior to 2 mg dose in achieving (SRI)-4 response compared to placebo after 24 weeks. However, it is widely debated whether these efficacy outcomes outweigh the increased susceptibility to infections and other risks posed by a higher dose. Those in favor have argued that the side effect profile is similar to ones observed in previous SLE trials. Risk of serious infections stood at 6% in a Baricitinib 4 mg group, 6% in a belimumab group, and 5% in a control group. Therefore, the risk and benefit of 4 mg Baricitinib for SLE need to be further investigated in clinical trials^39^. The unmet needs in SLE treatment are multifaceted and encompass various aspects such as inadequate disease control, significant burden of symptoms, and limited treatment options for refractory cases. One notable unmet need is the management of disease activity while minimizing treatment-related toxicity. Current treatment approaches often rely on glucocorticoids and immunosuppressive agents, which can lead to long-term adverse effects such as infections, organ damage, and metabolic complications. There is a pressing need for therapies that effectively control disease activity while minimizing these risks^40^.

### Limitations

Our meta-analysis pooled data from the three most recent randomized clinical trials investigating Baricitinib for SLE. However, the inherent limitations of the included studies should be taken into account when reviewing the findings. First, the relatively small number of included RCTs limits the generalizability of the findings. Second, the trial SLE-BRAVE-II ended abruptly, and only initial data were provided, which impacted the generalizability of the data. Third, a history of steroid and immunosuppressive therapies use can potentially act as a confounding factor which results in higher placebo rates effect size compared to the intervention^35^. This confounding effect is, however, not unique to Baricitinib but encompasses other SLE treatments, including ustekinumab trials (LOTUS)^39–41^. Fourth, SLE is a complex disease, and the selection of patients impacts the results^41^. A homogenous patient population with a smaller sample size and study duration may overestimate our results^42^. Fifth, the entire spectrum of SLE disease manifestations was not captured as both SLE-BRAVE-I and II excluding severe active renal and central nervous system diseases. Lastly, although population subgroups were analyzed in our meta-analysis, the individual patient-level data were not, which in our opinion, can allow improved insights into the assessment of treatment effects across varying patient profiles by identifying and minimizing potential bias.

### Implications for future research

It is hypothesized that a longer follow-up period would be better to measure the treatment response and identify potential adverse effects by taking multiple measurements instead of a single time cutoff. The present trials have only analyzed effects at 24 weeks and 52 weeks. In this regard, the SLE-BRAVE-X phase III SLE long-term extension trial is an ongoing trial planned to assess the long-term effectiveness and tolerability of Baricitinib for a three-year duration in adults who successfully underwent SLE-BRAVE-I or II. Supplementary studies are required to ascertain the optimal dosing and treatment duration of Baricitinib in SLE patients. Additionally, while designing protocols for future trials, strict recruitment criteria should be adhered to ensure trials’ success, as variations in baseline measures and patient characteristics can skew the findings. Lastly, based on the above discussion, appropriate outcome measurement should be used. Many investigators have attempted to unravel the best measure for SLE disease activity^43^. In the BLISS-52 study, the unique indicator LuMOS score differentiated between placebo and belimumab groups more efficiently than SRI-4. The LuMOS framework benchmarked the response via an elevated C4 complement level (mg/dL), diminished anti-double-strand DNA titers (IU/dl), a 4-point decrement on the SELENA-SLEDAI (yes/no), and changes in BILAG scores for organ system symptoms. As a result, it provides a thorough assessment compared to other existing markers and thus may be incorporated into the design of future trials.

## Conclusions

Baricitinib shows promising results in improving SRI-4 response, achieving a greater than or equal to 4-point improvement in SLEDAI-2K scores, and enhancing joint indices compared to placebo. However, the study highlights an increased incidence of serious adverse events, particularly a higher risk of serious infections associated with the 4 mg dose. These findings suggest that while Baricitinib could be an effective option for SLE management, its use should be approached with caution. Further long-term studies are essential to confirm these results and fully assess the safety and efficacy of Baricitinib, including the potential risks associated with higher doses, to guide clinical decision-making effectively.

## Ethical approval

Not applicable.

## Consent

Not applicable.

## Source of funding

Not applicable.

## Author contribution

M.A. conceived the idea. M.A. and O.S. designed the research workflow. M.A. and O.S. searched the databases. I.G., K.N.E. and M.A.E. screened the retrieved records, extracted relevant data, assessed the quality of evidence, and O.S. resolved the conflicts. A.R. performed the analysis. M.A., O.S., K.N.E., and U.A. wrote the final manuscript. B.A. supervised the project. All authors have read and agreed to the final version of the manuscript.

## Conflicts of interest disclosure

The authors declare no conflict of interest.

## Research registration unique identifying number (UIN)

Registered on PROSPERO, ID:CRD42023413579.

https://www.crd.york.ac.uk/prospero/display_record.php?RecordID=413579

## Guarantor

Basel Abdelazeem.

## Data availability statement

Not applicable.

## Provenance and peer review

Not commissioned, externally peer-reviewed.

## Supplementary Material
